# Individual Differences in Vicarious Pain Perception Linked to Heightened Socially Elicited Emotional States

**DOI:** 10.3389/fpsyg.2018.02355

**Published:** 2018-12-04

**Authors:** Vanessa Botan, Natalie C. Bowling, Michael J. Banissy, Hugo Critchley, Jamie Ward

**Affiliations:** ^1^School of Psychology, University of Sussex, Brighton, United Kingdom; ^2^Sackler Centre for Consciousness Science, Brighton, United Kingdom; ^3^Department of Psychology, Goldsmiths, University of London, London, United Kingdom; ^4^Brighton and Sussex Medical School, London, United Kingdom

**Keywords:** vicarious pain, affective empathy, cognitive empathy, individual differences in pain perception, self-other distinction

## Abstract

For some people (vicarious pain responders), seeing others in pain is experienced as pain felt on their own body and this has been linked to differences in the neurocognitive mechanisms that support empathy. Given that empathy is not a unitary construct, the aim of this study was to establish which empathic traits are more pronounced in vicarious pain responders. The Vicarious Pain Questionnaire (VPQ) was used to divide participants into three groups: (1) non-responders (people who report no pain when seeing someone else experiencing physical pain), (2) sensory-localized responders (report sensory qualities and a localized feeling of pain) and (3) affective-general responders (report a generalized and emotional feeling of pain). Participants completed a series of questionnaires including the Interpersonal Reactivity Index (IRI), the Empathy Quotient (EQ), the Helping Attitudes Scale (HAS), and the Emotional Contagion Scale (ECS) as well as The Individualism – Collectivism Interpersonal Assessment Inventory (ICIAI) and a self-other association task. Both groups of vicarious pain responders showed significantly greater emotional contagion and reactivity, but there was no evidence for differences in other empathic traits or self-other associations. Subsequently, the variables were grouped by a factor analysis and three main latent variables were identified. Vicarious pain responders showed greater socially elicited emotional states which included the ECS, the Emotional Reactivity Subscale of EQ and the HAS. These results show that consciously feeling the physical pain of another is mainly linked to heightened emotional contagion and reactivity which together with the HAS loaded on the socially elicited emotional states factor indicating that, in our population, these differences lead to a more helpful rather than avoidant behavior.

## Introduction

Some people automatically experience and re-create the physical pain of others on their own body and this has been known as vicarious pain responses or synaesthesia for pain (Fitzgibbon et al., 2010). Vicarious pain responses are mainly attributed to shared representations of self and other and supported by overlapping neuronal mechanisms of self-other pain processing (Lamm et al., 2011). Moreover, specific functional and structural neuronal patterns have been distinguished in populations characterized by conscious vicarious pain responses (Grice-Jackson et al., 2017).

In our past work, we developed the vicarious pain questionnaire (VPQ; Grice-Jackson et al., 2017) which separates participants into three categories when they observe the *physical* pain of others: (1) non-responders (report no pain when watching a video with someone else experiencing physical pain), (2) sensory-localized responders (report a localized feeling of pain in the same location as the person in the video) and (3) general-affective responders (report a generalized and emotional feeling of pain). The last two categories have been previously referred to as pain-responders (Derbyshire et al., 2013). Moreover, the sensory-localized group displays a capacity of mirroring the pain of another on oneself in a fashion similar to the tactile mirroring encountered in mirror-touch synaesthetes (Ward and Banissy, 2015). In the present study, we further investigate how individual differences in vicarious pain perception are linked to both affective and cognitive empathic traits.

A common link has been drawn in the literature between simulating the pain of others and empathy – the capacity to share and understand the emotional states of the others (de Vignemont and Singer, 2006; Lockwood, 2016). Importantly, empathy is not a unitary construct; it implies various components including affective empathy such as emotional contagion or emotional reactivity, cognitive empathy also referred to as Theory of Mind (ToM) or perspective taking, and compassionate empathy or empathic concern which can be associated with the action to help and alleviate other’s suffering (Bernhardt and Singer, 2012). Vicarious pain responses seem to have both a strong affective empathic component since they involve the representation of the painful emotional state of the other but also a cognitive/compassionate component. It is not clear yet to which extent feeling the physical pain of another benefits or impairs social interactions since the affective aspect of empathy is a fundamental process that allows recognizing and simulating others’ emotional states, but it does not necessarily require a cognitive understanding of their states (Bird and Viding, 2014). Vicarious pain responses seem to be mainly associated with an emotional reaction toward others’ states and previous research has indicated that individuals reporting conscious vicarious sensations, such as mirror touch synaesthetes (MTS), are more likely to score higher on the emotional reactivity subscale of the Empathy Quotient (EQ) but not on the other subscales (social skills and cognitive empathy) (Banissy and Ward, 2007). In this study, we use both the Emotional Reactivity scale of the EQ and, for the first time, the Emotional Contagion questionnaire to further investigate their association with vicarious pain responses.

There is still a debate regarding the extent to which emotional contagion and reactivity are related to empathy *per se*. For instance, Bird and Viding (2014) highlight that emotional contagion is a precursor of empathy and not an intrinsic component since empathy needs a clear distinction between self and other to occur. Moreover, a complete overlap between self and other representations would produce distress and impair the ability to switch between self and other perspectives (Lockwood, 2016). Thus, it is not clear whether strong emotional reactivity, as previously witnessed in vicarious perception, leads to empathic concern and altruistic behavior or, on the other hand, to personal distress and socially avoidant behaviors. It has been reported that higher levels of affective empathy lead to altruistic/pro-social behavior (Batson et al., 1981, 1997) and that pain intensity ratings correlate with higher empathic traits (Lamm et al., 2007). However, higher levels of personal distress can also be triggered when witnessing other’s pain especially if this is accompanied by a negative outcome (Lamm et al., 2007). As such there is likely to be a fine balance between the extent to which one can tune in to the feelings of others, and also the extent to which one can tune it out (using emotional regulation) to guard against personal distress.

Previous research has shown that self-other control (the ability to switch focus on information relevant to oneself or relevant to another person) improves performance in social cognitive domains. For instance, increased motor self-other control results in an increased vicarious pain perception (as measured by corticospinal activity and subjective ratings) and self-reported empathy in typical adults (de Guzman et al., 2015). This is in line with theoretical models of empathy suggesting that interactions between self-other control and vicarious perception may explain individual differences in empathy (e.g., Bird and Viding, 2014), which could perhaps be extended to those studied here. To date, few studies have studied self-other mechanisms in conscious vicarious pain responders (e.g., Derbyshire et al., 2013). Addressing this gap can enable a greater understanding of the structure of empathy (e.g., Bird and Viding, 2014; Ward and Banissy, 2015), including how individual differences in pain perception affect social cognition (e.g., Happé et al., 2017).

To identify which empathic traits vary in vicarious pain responders, we used a series of questionnaires looking at all these dimensions in the three different groups of people, recruited from the neurotypical population, but classified according to the VPQ. The groups are the independent variable. The dependent measures were: emotional contagion scale (ECS), the helping attitudes scale (HAS), the interpersonal reactivity index (IRI) and the EQ. These measures were employed to touch on all aspects of empathy from basic emotional contagion to motivational/compassionate empathy, including cognitive and affective aspects of empathy. Notably, most people do not manifest their compassion equally and they tend to favor those who are close to them (e.g., family, partners) and their ingroup, over strangers and out-groups. This also applies to measures relating to vicarious pain (Avenanti et al., 2010; Hein et al., 2010) and suggests a form of control mechanism by which people gate their empathic responses according to the degree to which others are self-related. For instance, family closeness is the strongest followed by closeness toward friends, colleagues and finally strangers (Matsumoto et al., 1997). As such, we tested whether vicarious pain responders show a different pattern (e.g., treating strangers like family) that might give rise to a different empathic response. We investigated the possible differences in the degree of social closeness and self- saliency in vicarious pain responders using the individualism-collectivism attitudes questionnaire (Matsumoto et al., 1997) and an abstract self-other association task (Sui et al., 2012). Sui et al. (2012) showed how people have faster reaction times when responding to an association made between self and an abstract shape than between another person (friend or stranger) and an abstract shape. These results support the idea that the self is prioritized, and this also seems to vary with cultural differences (Sui et al., 2009). We would expect the self not be as prioritized in vicarious pain responders and a linear trend in reaction times showing that this population treats unknown others as close ones or as self.

## Materials and Methods

### Participants

A total of 125 participants (mean age = 20.89, *SD* = 3.34; 104 females) completed the study. Participants were recruited via email invitation or via SONA from Sussex University and Goldsmiths, University of London. Each participant had previously completed the VPQ online via Bristol Online Survey (BOS) and were divided into three groups: controls (C), sensory-localized (S/L) and affective-general (A/G). The three groups were derived from a cluster analysis of a much larger dataset of participants who have completed the VPQ (Aged 18–60 years, *M* = 20.42 ± 4.16 SD, 297 Males, 759 Females). In order to obtain a medium effect size (0.5), with α err = 0.05, we aimed to recruit at least 30 participants in each group. Overall, there were 68 participants classed as controls, i.e., non- responders (mean age = 20.37, *SD* = 3.26, 58 females), 37 participants classed as S/L responders (mean age = 21.81, *SD* = 3.67, 29 females) and 21 participants classed as A/G responders (mean age = 21.00, 173 *SD* = 2.76, 17 females). The groups did not differ by age [*F*(2,124) = 2.241, *p* = 0.111, η^2^ = 0.035] or gender (χ^2^ = 0.469, *p* = 0.791). All participants completed the questionnaires: *EC, EQ, IRI, HAS*, and *ICIAI* (controls: *N* = 68 S/L: *N* = 37, A/G: *N* = 21). Due to technical issues, not all participants completed the *self- other association task* (controls: *N* = 55, S/L: *N* = 25, A/G: *N* = 16). Ethical approval was obtained from the Science and Technology Research Ethics Committee of the University of Sussex and all participants offered their written informed consent at the beginning of the study using an online form.

### Measures

#### Vicarious Pain Questionnaire

“The VPQ is comprised of 16 videos (no audio) of people experiencing physical pain (e.g., falls, sports injuries, injections), each video lasting for approximately 10 s (Grice-Jackson et al., 2017). After each video, participants were questioned about their experience. First, participants were asked if they experienced a bodily sensation of pain while viewing the video (yes/no). If the answer was “yes,” participants were asked to describe their pain by answering three more questions about their experience: (1) how intense their pain experience was (1–10 Likert scale, 1 = very mild pain, 10 = highly intense pain); (2) if and where they localized the pain, answering options were either “localized to the same point as the observed pain in the video,” “localized but not to the same point,” and “a general/non-localizable experience of pain”; (3) to select pain adjectives from a list that best described their vicarious pain experience (10 sensory descriptors such as “tingling,” “burning,” “stinging,” 10 affective descriptors such as “nauseating,” “grueling,” “aversive” and three cognitive-evaluative descriptors “brief,” “rhythmic,” “constant”). All these answers were used to generate the three variables that were entered the two-step cluster analysis (i.e., pain intensity, localized-generalized responses, and sensory – affective responses) which subsequently generated the three groups (for further details see Botan et al., 2018).”

#### Emotional Contagion Scale

The ECS (Doherty, 1997) is a 15-item self-reported unidimensional scale, with high reliability (Cronbach’s α = 0.90) which assesses the susceptibility to others’ emotions. The ECS consists of five basic emotions: love, happiness, sadness, anger, and fear. Each emotion is represented by three items (e.g.,*“If someone I’m talking with begins to cry,” “I get teary-eyed”* or *“Being with a happy person picks me up when I’m feeling down”)* that are scored on a 5-point Likert scales from 1 – *not at all* to 5 – *always*, with a higher score indicating higher emotional contagion.

#### Empathy Quotient

*A* short 15-item version of the EQ (Muncer and Ling, 2006) was used comprising five items for each of the three subscales: Social Skills (SS) (e.g., “*I find it had to know what to do in a social situation”*) (Cronbach’s α = 0.57), Cognitive Empathy (CE) (e.g., “*I am good at predicting how someone will feel”*) (Cronbach’s α = 0.74), and Emotional Reactivity (ER) (e.g., “*Seeing people cry does not really affect me”*) (Cronbach’s α = 0.63). Participants gave their responses on a 4-point Likert scale, ranging from 1 – *strongly disagree* to 4 – *strongly agree*.

#### Interpersonal Reactivity Index

The Interpersonal Reactivity Index, or IRI (Davis, 1983), is a multidimensional scale, comprised of 28 items divided into four subscales. The subscales are Perspective Taking (PT) (e.g., “*I try to look at everybody’s side of a disagreement before I make a decision”.)*, Fantasy Scale (FS) (e.g., “*After seeing a play or movie, I have felt as though I were one of the characters.”)*, Empathic Concern (EC) (e.g., “*I am often quite touched by things that I see happen.”)*, and Personal Distress (PD) (e.g., “*When I see someone who badly needs help in an emergency, I go to pieces”*). Each subscale consists of seven items and responses are given on a five-point scale 0 – *does not describe me very well* to 4-*describes me very well*.

#### Helping Attitudes Scale

The Helping Attitude Scale (Nickell, 1998) is a self-report unidimensional measure of pro-social and helping tendencies with good internal consistency (Cronbach’s α = 0.869). It comprises 20 items scored on a 5-point Likert scale (1 = *strongly disagree* to 5 = *strongly agree*). Examples of items are: “*Helping others is usually a waste of time;” “When given the opportunity, I enjoy aiding others who are in need;” “It feels wonderful to assist others in need*.”

#### The Individualism – Collectivism Interpersonal Assessment Inventory (ICIAI)

The Individualism – Collectivism Interpersonal Assessment Inventory (ICIAI) (Matsumoto et al., 1997) assesses values (Part 1) and behaviors (Part 2) when interacting with others. It takes into account the degree of closeness with the other in four relationship groups: family, friends, colleagues and strangers. We were mainly interested in behaviors and so we only used the second part of the questionnaire. Participants scored from 0 = *never* to 6 = *all the time* how much they engaged in each of the mentioned behaviors toward each of the four relationship groups. The reliability of the questionnaire is high with Cronbach’s α = 0.90. The questionnaire contains 19 items and examples are: “*Maintain self-control toward them; Share blame for their failures; Sacrifice your possessions for them; Respect them” etc.*

#### Self-Other Association Task

The self-other association task (Sui et al., 2012) requires participants to respond to an association between a geometric shape (triangle, square, or circle) and a label (self, a named best friend, or an unfamiliar person). Participants were first asked to name a best friend and the time- period they had known each other for. Then each of the three geometrical shapes was randomly associated to a label (e.g., *you are a circle, the stated friend is a triangle, and a stranger is a square*). In the matching phase, the participants had to judge if the match shapes- label pairings was correct. A pairing of a shape and a label (e.g., Δ – *stranger*) was presented for 500 ms. The pairing was generated at random and it could conform to the initial instruction which associated each shape to a specific label, or it could be a recombination of a label with a different shape. Immediately after, participants were expected to judge of the association was correct or not. Participants first performed a practice phase containing 20 trials when they were given written feedback (correct or incorrect) followed by three blocks of 120 trials. Thus, there were 60 trials in each condition across all blocks (self-matched, self-nonmatching, familiar-matched, familiar-nonmatching, unfamiliar- matched, and unfamiliar-nonmatching). Reactions times were recorded and analyzed as dependent variable in a mixed model ANOVA.

### Procedure

The questionnaires were administered via Bristol Online Survey (BOS), an online software for collecting questionnaire data. The self-other association task was run via Inquisit^1^, an online survey for collecting both questionnaire and tasks data. Participants filled in the questionnaires and, subsequently, they were re-directed to the task. The study took approximately 40 min (30 min for questionnaires and 10 min for the task). All questionnaires were completed in the same order (as outlined above), so groups were matched in this regard.

### Statistical Analyses

Analyses of variance (one-way ANOVAs) were used to establish differences between groups on each questionnaire. Mixed models analyses of variance were run on the ICIAI (3 groups × 4 conditions ANOVAs) and on the self-other association tasks (3 groups × 3 conditions ANOVAs). Variables were treated as continuous and the great majority of them were normally distributed as shown by Shapiro–Wilk tests and histograms. Normality assumptions were violated only in the following cases: controls [IRI-EC (*p* = 0.01) and ICIAI family (*p* = 0.01) and colleagues (*p* = 0.04)]; S/L [EQ-CE (*p* = 0.02), IRI-EC (*p* = 0.01)]. For these cases, Kruskal–Wallis non-parametric tests were run, re-confirming the results (see Supplementary Result S1). All analyses were run in SPSS separately for each measure and test-wise Bonferroni confidence interval adjustment was used for comparisons of main effects. Both Games-Howell and Hochberg’s GT2 *post hoc* tests for different sample sizes were run (Field, 2013). Effect sizes (Cohen’s *d*) were also calculated and reported in Supplementary Result S2. A principal axis factor analysis (FA) was conducted on nine variables (IRI-EC, IRI-FS, IRI-PT, IRI-PD; EQ- SS, EQ-CE, EQ-ER; ECS; HAS) which generated three latent variables. Data for this analysis has been made available in Supplementary Table S1. Analyses of multivariance (MANOVAs) were used to establish differences between groups on the three latent variables.

## Results

### Between Group Differences: One-Way ANOVAs

There were significant group differences on ECS [*F*(2,122) = 5.281, *p* = 0.006, η^2^ = 0.08], both sensory-localized and affective-general groups scored higher than controls (S/L: *p* = 0.028, A/G: *p* = 0.034) but did not differing from each other (*p* = 0.915). There was a significant group difference on the emotional reactivity subscale of the EQ [*F*(2,122) = 5.247, *p* = 0.007, η^2^ = 0.08], with both sensory-localized and affective- general groups scored higher than controls (S/L: *p* = 0.02, A/G: *p* = 0.05) but not different from each other (*p* = 0.99). None of the other subscales of the EQ showed differences between groups: Cognitive Empathy [*F*(2,122) = 2.297, *p* = 0.105, η^2^ = 0.031] and SSs [*F*(2,122) = 0.370, *p* = 0.695, η^2^ = 0.006].

**FIGURE 1:**
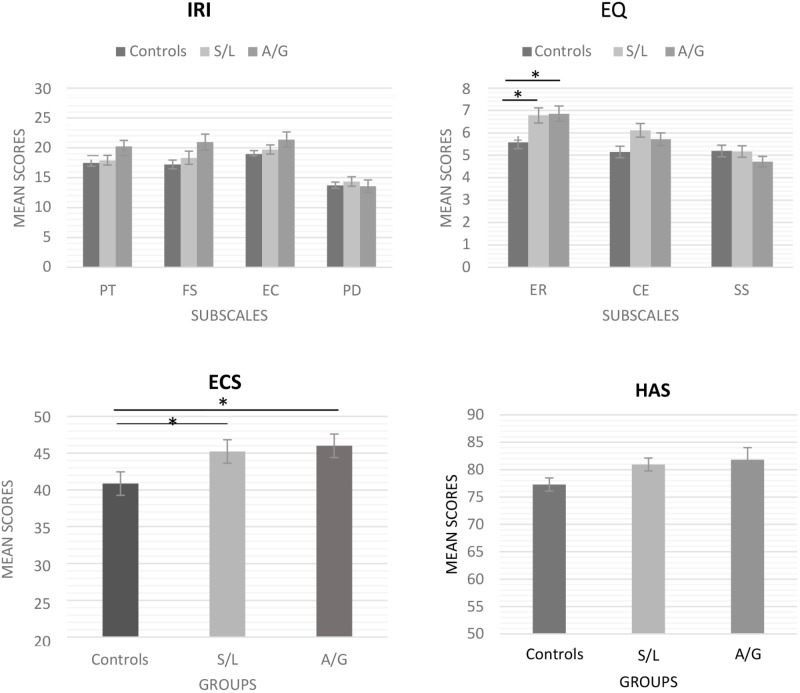
IRI, EQ, ECS, and HAS scores. S/L, sensory-localized; A/G, affective-general. Both S/L and A/G scored higher on emotional contagion (ECS) and emotional reactivity (EQ-ER) than controls but not on cognitive empathy (EQ-CE) or social skills (EQ-SS) subscales. No significant differences were found on IRI and HAS. Error bars indicate ±1 SE. ^∗^*p* < 0.05.

The results of the questionnaire measures are summarized in Figure 1.

IRI scores did not show any significant differences on Personal Distress [*F*(2,122) = 0.296, *p* = 0.744, η^2^ = 0.005] or in empathic concern [*F*(2,122) = 0.296, *p* = 0.141, η^2^ = 0.032] but there was a trend toward increased scores in vicarious perceivers for perspective taking [*F*(2,122) = 2.930, *p* = 0.057, η^2^ = 0.046] and fantasy [*F*(2,122) = 2.981, *p* = 0.054, η^2^ = 0.047] subscales. The HAS revealed no significant differences between groups [*F*(2,122) = 2.576, *p* = 0.08, η^2^ = 0.041].

### Between Group Differences: Factor Analysis and MANOVAs

A principal axis factor analysis (FA) was conducted on nine variables with oblique rotation (direct oblimin). The Kaiser–Meyer–Olkin measure verified the sampling adequacy for the analysis, KMO = 0.741 and all KMO values for individual variables were greater than the acceptable limit of 0.5 (Kaiser, 1974). An initial analysis was run to obtain eigenvalues for each factor in the data. Three factors had eigenvalues over Kaiser’s criterion of 1 and in combination explained 64.8% of the variance. The scree plot was ambiguous and showed inflections that would justify retaining both two or three factors (Field, 2013). We retained three factors because of the convergence of the scree plot and Kaiser’s criterion on this value. IRI-EC, IRI-PT, and IRI-FS clustered on factor 1, EQ-SS, EQ-CE, and IRI-PD clustered on factor 2, EQ-ER, ECS, and HAS clustered on factor three. Thus, we distinguished between three underlying latent variables: interpersonal and imaginary abilities (Factor 1), low emotion regulation (Factor 2), and socially elicited emotional states (Factor 3). The results can be seen on Table 1.

**Table 1:** Factor analysis results.

Rotated factor loadings			
Variable	Interpersonal and imaginary abilities	Low emotion regulation	Socially elicited emotional states
IRI_EC	**0.81**	0.03	−0.01
IRI_FS	**0.79**	−0.06	−0.18
IRI_PT	**0.75**	0.06	0.25
EQ_CE	0.10	**−0.77**	0.23
EQ_SS	0.05	**−0.74**	0.13
IRI_PD	0.20	**0.64**	0.45
ECS	−0.22	0.03	**0.88**
EQ_ER	0.25	−0.22	**0.69**
HAS	0.19	−0.23	**0.56**
**Eigenvalues**	**3.12**	**1.50**	**1.20**
**% of variance**	**34.67**	**16.64**	**13.35**

*Values in bold indicate the highest loadings on each factor.*

The three latent variables identified by FA were included in a multivariate analysis of variance (MANOVA). All variables respected the assumption of normality, the only exception being the interpersonal and imaginary ability variable in the A/G (Shapiro–Wilk test, *p* = 0.04). Two outliers were excluded from the A/G group and the Box’s test confirmed the assumption of equal covariance (*p* = 0.08). Pillai’s trace multivariate test revealed significant effect *F*(3,238) = 3.663, *p* = 0.002 and separate univariate tests showed that there was a significant differences between groups on interpersonal and imaginary abilities *F*(2,129) = 4.781, *p* = 0.01 and on socially elicited emotional states *F*(2,120) = 8.122, *p* < 0.001 but not on low emotion regulation *F*(2,120) = 1.181, *p* = 0.311. *Post hoc* tests indicated that the A/G group scored higher than controls on the interpersonal and imaginary ability (*p* = 0.007) but there was no difference between S/L and controls (*p* = 0.66). Both S/L and A/G groups scored higher on socially elicited emotional states (*p* = 0.008, *p* = 0.003 respectively). There were no differences between the two groups or between the two groups and controls in emotion regulation (S/L vs. C, *p* = 0.35; A/G vs. C, *p* = 0.99; S/L vs. A/G, *p* = 0.67).

### Self-Other Associations

**FIGURE 2:**
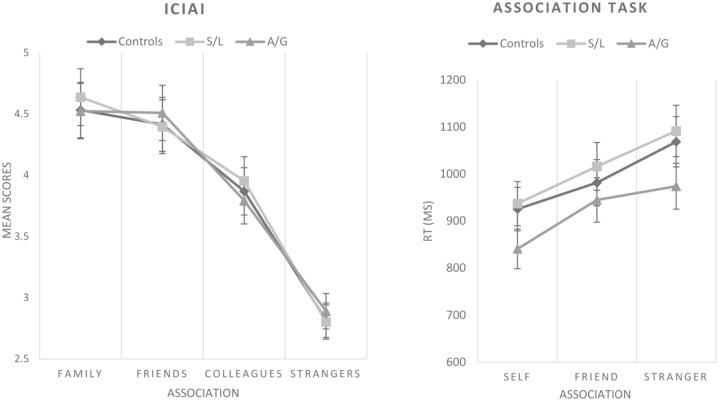
ICIAI and self-other association task results. S/L, sensory-localized; A/G, general affective. The effects of closeness appear both in subjective scores and in task reaction times but not as an effect of group. All groups show a similar trend in RTs to the self-other association. Error bars indicate ±1 SE.

The Individualism-Collectivism Interpersonal Assessment (ICIAI) was analyzed as a 3 × 3 mixed ANOVA contrasting group (control, S/L, A/G) and closeness (family, friend, stranger). There was a main effect of closeness [*F*(3,122) = 246.405, *p* < 0.001, η^2^ = 0.669] but there was no main effect of group [*F*(2,122) = 0.619, *p* = 0.941 η^2^ = 0.001] or interaction [*F*(6,122) = 0.536, *p* = 0.949, η^2^ = 0.003]. At a behavioral level, the self-other association task was also analyzed as a 3 × 3 mixed ANOVA contrasting group (control, S/L, A/G) and closeness (self, friend, stranger) on response times to correctly endorse matching pairs (see Sui et al., 2012). There was a significant effect of closeness [*F*(2,94) = 29.818, *p* < 0.001, η^2^ = 0.241] but no main effect of group [*F*(2,94) = 0.600, *p* = 0.551, η^2^ = 0.013] and no interaction [*F*(4,1.940) = 0.134, *p* = 0.781, η^2^ = 0.009]. Correlations between the questionnaire empathic measures and task RTs were run on the entire sample but there were no significant results (see Figure 2).

All together, these results indicate that vicarious pain responders have heightened socially elicited emotional states but none of the groups differ from controls on emotion regulation and neither on subjective (as measured by ICIAI) or objective (as measured by the task) self-other associations. Overall, vicarious pain responders seem to have higher emotional responsiveness than non- responders but no differences in emotion regulation or their reports with others.

## Discussion

The capacity to co-represent the feelings of other people has a central role in most theoretical accounts of empathy (de Vignemont and Singer, 2006; Lockwood, 2016). However, the mechanism by which this occurs remains under debate as does its relationship to social behavior. For instance, whilst empathy may underpin acts of compassion (Singer and Klimecki, 2014) it has also been claimed that too much empathy can be detrimental (Bloom, 2017). In the present study, we took advantage of a recently reported individual difference in the neurotypical population; namely, the extent to which people report consciously feeling pain when observing other people in pain. Some people report feeling the pain of others either localized on the corresponding part of their own body (Sensory-Localized responders, S/L) or a non-localized, more general body feeling (Affective-General responders, A/G). However, the majority of people report no conscious feelings of pain: they either have an implicit simulation or possibly do not simulate the pain of others. In this study, we assessed for the first time how these individual differences in vicarious pain are linked to differences in various dimensions of empathy and relationships with others. We employed a series of questionnaires to test between groups differences and, given the multitude of variables used, we also ran a factor analysis which showed that there were three underlying latent variables: socially elicited emotional states (ECS, EQ-ER, and HAS), interpersonal and imaginary abilities (IRI: PT, EC and FT), and low emotion regulation (EQ – SS, EQ-CE, IRI-PD).

### Socially Elicited Emotional States

Both S/L and A/G vicarious pain responders report a greater perception of socially elicited emotional states. This suggests that vicarious pain perception is probably just one trait of a much broader phenotype in conscious vicarious pain responders (including emotion contagion as well as the defining symptom of ‘pain contagion’). Moreover, the socially elicited emotional states variable includes both measures of emotional responsivity and helping behaviors. HAS loaded on the same latent variable as emotional reactivity/contagion indicating that higher responsiveness to others’ emotions is linked to a helpful behavior rather than an avoidant one. This may be explained by the fact that that helping someone leads to a change in the emotional state of the helper, as some of the HAS items point out (e.g., It feels wonderful to assist others in need) which would be more noticeable in people with elevated emotional contagion. Since there were no differences recorded in the other variables, this behavior may also be mediated by their intact social-cognitive skills and their ability to distinguish between self and other (Bird and Viding, 2014).

### Low Emotion Regulation

Despite having shared representations of pain and enhanced affective empathy, vicarious pain responders did not report enhanced SSs and neither personal distress. It seems like these behaviors are neither impaired nor stimulated by strong emotional responses as previously stated by Bloom (2017) (N.B. we only recorded general, trait attitudes in this study and not immediate responses to painful stimulation). Interestingly, social and cognitive skills (the two EQ subscales) and Personal Distress (the IRI subscale) all loaded on the same factor showing that the more Personal Distress someone reports, the lower his/her social – cognitive skills are. Thus, impaired social- cognitive skills lead to higher levels of personal distress and, together, to low emotion regulation which seems to be mainly linked to poor social-cognitive skills rather than high emotional responsiveness. Vicarious pain responders are characterized by higher socially elicited emotional states, but they have typical social-cognitive skills and emotion regulation suggesting that the mechanisms for these different empathic qualities could be segregated and function independently, but the mechanism is not yet fully understood. Reporting feeling the pain of others does not seem to impact in any way their ability to relate to the other or their levels of personal distress. In the wider literature, symptoms such as emotional contagion are regarded as developmental precursors of empathy, which are diminished as emotional regulation mechanisms mature (Thompson, 1991; Eisenberg, 2000). People with vicarious pain appear to have retained a high capacity for emotional contagion but without reporting a concomitant problem in regulating or coping with these symptoms. Osborn and Derbyshire (2010) also reported that, in vicarious pain responders, there was no correlation between vicarious pain intensity and Personal Distress. The fact that vicarious pain perceivers do not have higher levels of Personal Distress may be due to habituation to pain which sometimes is noticed in response to frequent exposure to pain (Bingel et al., 2007) or to the fact that they developed a response mechanism toward occurrence of pain. Thus, a testable prediction is that these populations would have better emotional regulation which would be recorded in both questionnaires and physiological measures such as heart-rate variability (Appelhans and Luecken, 2006) and would shed more light on bodily and emotional processing in vicarious pain responders.

### Interpersonal and Imaginary Abilities

Three of the scales of the IRI (Empathic Concern, Perspective Taking, Fantasy) were found to be associated together, and the Affective/General group scored significantly higher on this factor. These measures tend to reflect a more deliberate empathic style (e.g., choosing to take another person’s perspective) than the emotional contagion/reactivity measures already discussed (which were elevated in both responder groups and with larger effect sizes). Further studies combining behavioral and neuroscientific measures in these groups are needed to establish what underpins this. Previous research indicated that individual differences in perspective taking (PT subscale of IRI) and empathic concern (EC subscale of IRI) influence the feeling of being touched (Bolognini et al., 2013b). Experimentally induced excitability over somatosensory cortex can elicit synaesthetic mirror-touch phenomena (Bolognini et al., 2013a,b, but also see Bowling and Banissy, 2017) a phenomenon similar to mirror-pain responses of the S/L group. However, whilst these studies found that the IRI predicted tactile sensations in their sample (likely comprising non-responders), the IRI was not elevated in the S/L group (see Ward et al., 2018). With regards to perspective taking, Derbyshire et al. (2013) found that vicarious pain responders were more influenced by the visual perspective of an avatar when judging from their own viewpoint (but they did not distinguish between different kinds of responders). Bucchioni et al., 2016 showed that motor-evoked responses are inhibited more when participants observe the pain from a first - person perspective than from a third-person perspective (hand that receives the pain is rotated at 180°). If vicarious pain responders are more influenced by a third perspective, then we would expect them to show greater inhibition of motor evoked responses in this condition too.

### Self-Other Associations

There were no differences in self-other associations between vicarious pain responders and controls. In both the subjective (ICIAI questionnaire) and objective (self-other association task) measures, we would have expected a linear trend showing that vicarious pain responders treated unknown others as close ones or as self. The results did not confirm this hypothesis. The ICIAI has a strong cultural component whilst the self-other association task requires an abstract association and recorded reaction times to congruent or incongruent association between a geometrical shape and a label. The task mainly determines changes in perceptual saliency by employing various self- other associations and the use of self-associated labels. Importantly this type of task does not require participants to engage in online control of self-other representations. That is to say that participants do not have to co-represent themselves and others in the same trial because they are cued toward self or other, and thus it is unlikely that self or other are represented at the same time (i.e., only the self or other is represented, but not both). Prior work suggests that the online control of co- activated self-other representations is linked to empathy and associated brain networks including the rTPJ (e.g., Santiesteban et al., 2012, 2015; Sowden et al., 2015; Nobusako et al., 2017), but the ability to attribute mental states to the self or others does not tend to recruit this same brain network (e.g., Lombardo et al., 2010; Sui et al., 2013a,b). Given that individuals with conscious vicarious pain perception have been shown to differ in their neural profile within the rTPJ (Grice-Jackson et al., 2017) it perhaps more likely that they will differ on tasks that involve the online control of co-activated self-other representations, than tasks that tap into the ability to attribute states to the self or others via cues like the one used in the current investigation.

### Summary

Overall, our results indicate that vicarious pain responses are mainly linked to heightened socially elicited emotional states and we obtained no evidence for significant differences in emotion regulation or self-other associations. Moreover, differences in perspective taking and imaginative abilities were only recorded in the A/G group. These results further characterize vicarious pain responders and indicate that consciously feeling the physical pain of another is associated with heightened socially elicited emotional states but not with low emotion regulation. Thus, the heightened emotional responsiveness observed in vicarious pain responders is mainly associated with a helping rather than avoidant behavior and good emotion regulation could mediate this mechanism.

## Ethics Statement

This study was carried out in accordance with the recommendations of the Science and Technology Research Ethics Committee of the University of Sussex (C-REC). The protocol was approved by the Science and Technology Research Ethics Committee of the University of Sussex (C-REC). All subjects gave written informed consent in accordance with the Declaration of Helsinki.

## Author Contributions

JW and VB designed the study, collected the data, and wrote the paper. NB and MB collected the data and wrote the paper. HC wrote the paper.

## Conflict of Interest Statement

The authors declare that the research was conducted in the absence of any commercial or financial relationships that could be construed as a potential conflict of interest.

## Footnotes

1 http://www.millisecond.com
