# Supplementary material for: Individual Differences in Vicarious Pain Perception Linked to Heightened Socially Elicited Emotional States
Source: Front Psychol. 2018 Dec 4;9:2355. doi: 10.3389/fpsyg.2018.02355 (PMC6288400; doi:10.3389/fpsyg.2018.02355)
Supplement: Supplementary file 1 [file Data_Sheet_1.docx]

**Supplementary Results**

**S1. Non-parametric tests results.**

Kruskal-Wallis H test for measures that were not normally distributed re-confirmed the parametric test results regarding differences between groups: EQ-C ( χ^2^ = 5.061, p = 0.080), IRI-EC ( χ^2^ = 4.698, p = 0.095), ICIAI family ( χ^2^ = 0.710, p = 0.701) and colleagues ( χ^2^ = 1.284, p = 0.526).

**S2. Effect sizes**

**Figure 2** Effect sizes for EC, EQ subscales, IRI subscales and HAS for S/L and A/G when compared to controls. Medium effect sizes (d>0.5) were observed on EC and EQ-ER for both S/L and A/G and on IRI-PT and IRI-F only for A/G. All the other effect sizes were small.
